# Ocular surface indicators and biomarkers in chronic ocular graft-versus-host disease: a prospective cohort study

**DOI:** 10.1038/s41409-021-01254-5

**Published:** 2021-03-08

**Authors:** Alexandra A. Pietraszkiewicz, Debbie Payne, Maria Abraham, Angel Garced, Krishna C. Devarasetty, Megan Wall, Supriya M. Menezes, Sveti Ugarte, Filip Pirsl, Sencer Goklemez, Frederick L. Ferris, John Barrett, Minoo Battiwalla, Richard W. Childs, Steven Z. Pavletic, Rachel J. Bishop

**Affiliations:** 1 National Eye Institute, National Institutes of Health, Bethesda, MD USA; 2 The Emmes Company, LLC, Rockville, MD USA; 3 Allergan, Inc., Irvine, CA USA; 4 National Cancer Institute, Center for Cancer Research, National Institutes of Health, Bethesda, MD USA; 5 National Heart, Lung, and Blood Institute, National Institutes of Health, Bethesda, MD USA

**Keywords:** Diagnosis, Graft-versus-host disease, Risk factors, Signs and symptoms

## Abstract

This longitudinal cohort study compared ocular surface indicators in forty allogeneic hematopoietic stem cell transplant (HSCT) subjects with twenty healthy controls at baseline and identified changes in ocular graft-versus-host disease (oGVHD). Outcome measures included: Ocular Surface Disease Index (OSDI), tear osmolarity, Schirmer’s test, Oxford corneal staining score, tear break-up time (TBUT), and tear and serum biomarkers (IFN-γ, IL-10, MMP-9, IL-12, IL-13, IL-17α, IL-1β, IL-2, IL-4, IL-6, IL-8, CXCL10, MCP-1, MIP-1α, RANTES, TNF-α). At baseline the HSCT group had higher median Oxford corneal staining score (1.7 vs. 0.0; *P* < 0.0001), higher tear TNF-α (20.0 vs. 11.2 pg/mL; *P* < 0.0001), lower tear RANTES (70.4 vs. 190.2 pg/mL; *P* < 0.0001), higher serum IL-8 (10.2 vs. 4.5 pg/mL; *P* = 0.0008), and higher serum TNF-α (8.7 vs. 4.2 pg/mL; *P* < 0.0001). The incidence of oGVHD was 62% and associated changes included increased Oxford corneal staining score (4.6 vs. 1.8, *P* = 0.0001), decreased Schirmer’s test (3.0 vs. 10.0; *P* < 0.0001), and decreased TBUT (4.7 vs. 9.0 s; *P* = 0.0004). Baseline differences in ocular surface indicators suggest a tendency toward ocular dryness in individuals with hematologic disorders preparing for HSCT. Individuals who developed oGVHD showed changes in corneal staining score, Schirmer’s test, and TBUT.

## Introduction

Allogeneic Hematopoietic Stem Cell Transplantation (HSCT) is a potentially curative treatment for many hematologic diseases [1]. A major cause of morbidity and mortality following HSCT is graft-versus-host-disease (GVHD), a T-cell mediated immunologic response of the donor graft to host antigens [2]. The most important of these are human leukocyte antigens, found on virtually all nucleated cells [2]. Systemic chronic GVHD (cGVHD) occurs in 30–70% of transplant recipients; ocular manifestations occur in up to 90% of patients with cGVHD [1]. The main targets of disease in ocular chronic GVHD (oGVHD) are structures of the ocular surface, including the eyelids, lacrimal gland, conjunctiva, and cornea [1]. Fibrosis and destruction of ocular surface tissues result in the clinical signs and symptoms of keratoconjunctivitis sicca, blepharitis, and Meibomian gland dysfunction [1].

Baseline ophthalmological evaluation of patients undergoing HSCT has been recommended by the National Institutes of Health (NIH) Consensus Conference (2014), the First International Chronic Ocular GVHD Consensus Group (2013), and the German–Austrian–Swiss Consensus Conference (2012) [3–5]. Dry eye disease (DED), a multifactorial disorder characterized by loss of homeostasis of the tear film accompanied by ocular symptoms [6], occurs in ~50% of HSCT patients at baseline [7, 8], with alterations in ocular surface parameters developing after HSCT regardless of oGVHD diagnosis [9]. Studies have explored various ocular surface parameters [10] in an attempt to identify risk factors associated with the development of oGVHD and increased severity of oGVHD [11–14] and to more effectively understand the course of disease over time [15]. Tear and serum cytokines have been investigated in the hopes of establishing more reliable predictive, diagnostic, and prognostic biomarkers [16–18].

In this prospective study, 40 individuals with a hematologic disorder or malignancy who were scheduled to receive a HSCT and 20 healthy controls underwent comprehensive ophthalmologic evaluations including administration of the Ocular Surface Disease Index (OSDI) Questionnaire, a detailed ocular surface assessment, and tear and serum biomarker analysis. Baseline findings were compared between the groups. The HSCT cohort was followed over time to identify risk factors for and markers of oGVHD development. In addition, for those HCST patients who received at least one year of ophthalmic follow-up, comparison of ocular indicators and biomarkers was made between the month 12 and baseline visits.

## Subjects and methods

### Patients and healthy controls

The study followed the tenets of the Declaration of Helsinki and was approved by the Institutional Review Board of the National Eye Institute (NEI), NIH. Written, informed consent was obtained from each patient after explanation of the nature and possible consequences of the study. Since this was an exploratory study, no formal sample size estimation was performed. Participants were enrolled from HSCT treatment protocols at the National Cancer Institute (NCI) and the National Heart, Lung and Blood Institute (NHLBI) at NIH. Participants had to be at least 18 years of age and had to be scheduled for a HSCT under an NIH protocol. Participants had a range of primary diagnoses (Table 1). Exclusion criteria included a history of ocular problems that could interfere with the natural history of their response to treatment with HSCT (significant dry eye disease, use of cyclosporine eye drops in the past 30 days, conjunctival scarring, etc.), and known allergies to dilating or anesthetic eye drops.

**Table 1:** Baseline characteristics of patients and controls.

	Characteristic	Intended HSCT cohort (*n* = 40)^a^	Controls (*n* = 20)^a^
Age	Median (range)	40.5 (20–70)	38.5 (21–73)
	Mean	41.3	39.3
Sex	Male	21 (52.5%)	9/20 (45.0%)
	Female	19 (47.5%)	11/20 (55.0%)
Primary diagnosis	AA	8 (20%)	
	ALL	9 (22.5%)	
	AML	7 (17.5%)	
	APL	1 (2.5%)	
	CGD	1 (2.5%)	
	CLL	1 (2.5%)	
	Cutaneous Lymphoma	1 (2.5%)	
	Follicular Lymphoma	1 (2.5%)	
	HL	1 (2.5%)	
	Mantle Cell Lymphoma	1 (2.5%)	
	MDS	4 (10.0%)	
	MM	1 (2.5%)	
	Plasmocytic dendritic cell neoplasm	1 (2.5%)	
	SCA	3 (7.5%)	
Type of transplant (*n* = 36)^b^	HLA matched	32 (88.8%)	
	Unmatched	4 (11.1%)	
Total Body Irradiation	Yes	25 (69.4%)	
	Low-dose (<600 Gy)	6/25 (24.0%)	
	High-dose (≥600 Gy)	19/25 (76.0%)	
	No	11 (30.5%)	
Conditioning regimen	Myeloablative	20 (55.5%)	
	Non-Myeloablative	16 (44.4%)	
SteM cell source	Cord blood and peripheral blood	4 (11.1%)	
	Peripheral blood	32 (88.8%)	

^a^N corresponds to the number of participants.

^b^Four patients did not receive an HSCT.

*HSCT* hematopoietic stem cell transplant, *AA* aplastic anemia, *ALL* acute lymphoblastic leukemia, *AML* acute myeloid leukemia, *APL* acute promyelocytic anemia, *CGD* chronic granulomatous disease, *CLL* chronic lymphoblastic leukemia, *HL* hodgkin lymphoma, *MDS* myelodysplastic syndrome, *MM* multiple myeloma, *SCA* sickle cell anemia, *HLA* human leukocyte antigen, *Gy* gray.

The intended HSCT cohort included 40 individuals. However, four individuals did not receive a transplant and two died within one month of transplant; the remaining 34 individuals comprised the analytic HSCT cohort. Of those, 30 individuals had at least 12 months of follow-up, comprising the longitudinal HSCT cohort. Healthy controls (*n* = 20) were recruited through the NEI consult service (at NIH) and were at least 18 years of age without significant systemic or ocular disease.

### ClinicaL examination and sample collection

Participants underwent the following tests as part of their baseline and subsequent evaluations: medical and ophthalmic history, OSDI scoring, best-corrected visual acuity, tonometry, Schirmer’s test with anesthesia, tear break-up time (TBUT), a detailed anterior segment examination, conjunctival and corneal surface examinations including vital dye staining with lissamine green and fluorescein, and a dilated fundus examination. The anterior segment examination included evaluation of the lacrimal gland, eyelids including Meibomian glands, conjunctiva, cornea, anterior chamber, and iris. Corneal and conjunctival staining was graded using the Oxford scheme, with two modifications. First, a decimalized scale allowed graders optimal flexibility in assigning values to each segment (cornea, nasal conjunctiva, and temporal conjunctiva). Second, a value of zero indicated no punctate keratopathy, which differs from the standard grading scale which allows for a single punctate defect in the grade of zero. Each segment received a score of zero to five, with a total Oxford score of zero to 15. Tear fluid was collected and analyzed for osmolarity using the Tearlab Osmolarity System (San Diego, CA) and analyzed for biomarkers by Allergan, Inc. Serum obtained from blood samples (~5cc) was also analyzed for biomarkers (Allergan, Inc.).

Examinations were performed at baseline (between 10 and 112 days before HSCT), and at months 1, 3, 6, 9, and 12 after HSCT, with an optional extension to 18 and 24 months, and then annually for up to 5 years. Participants received all standard ocular therapies to manage dry eye and oGVHD as clinically indicated, including topical lubrication, topical cyclosporine, topical steroids, punctal occlusion, eyelid compresses and scrubs, and environmental modifications such as humidifiers. Participants were allowed to receive treatment from their home ophthalmologists throughout the study. Diagnosis of Dry Eye Disease (DED) was made according to the 2007 Report of the International Dry Eye Workshop (DEWS), the current guideline at time of study initiation [19]. Diagnosis of oGVHD was made according to the NIH consensus criteria (2014) [3]. Global scoring of chronic GVHD and organ-specific scoring were performed according to the same guidelines. For patients with DED at baseline, evidence of increased staining compared to baseline with a decrease in Schirmer’s test to a value between 6 and 10 mm, or a decrease in Schirmer’s test to below 5 mm was required for diagnosis of oGVHD.

### Statistical analysis

For tear and serum biomarker analyses, a Bonferroni-corrected type I error rate of 0.16% was used. For the remaining analyses, a type I error rate of 5% was used and no adjustments were made for multiplicity. The following analyses were performed:

- Comparison of baseline ocular factors and tear and serum biomarkers between the intended HSCT group (*n* = 40) and controls (*n* = 20) using a nonparametric Wilcoxon rank-sum test.
- Among those who developed oGVHD (*n* = 21): comparison of ocular factors and tear and serum biomarkers at time of diagnosis of oGVHD vs. baseline, using a Wilcoxon signed-rank test. This analysis did not account for varying times to diagnosis of oGVHD. If a participant was diagnosed with oGVHD in both eyes at the same visit, both eyes were included in the analysis. If they were diagnosed with oGVHD in one eye, even if the other eye was diagnosed at a later visit, only the first affected eye was included in the analysis. Unaffected eyes were not included in the analysis.
- Within the longitudinal HSCT group (*n* = 30): comparison of ocular factors and tear and serum biomarkers at month 12 vs. baseline, using a nonparametric Wilcoxon signed-rank test and a mixed-effect longitudinal model with time as a fixed effect and compound symmetry variance-covariance matrix.
- Receiver Operating Characteristic (ROC) Curve Analysis. Ocular factors and tear and serum biomarkers (a) at the time of diagnosis of oGVHD for patient eyes diagnosed with oGVHD, and (b) at the most recent measurements available for patient eyes not diagnosed with oGVHD, were used to plot the ROC curve and subsequently determine the optimal cutoff value for oGVHD diagnosis. The Area Under the ROC Curve (AUC) was evaluated for all ocular factors and tear and serum biomarkers for the diagnosis of oGVHD. The optimal cutoff was chosen as the value that maximized both sensitivity and specificity for those parameters with AUC > 0.7. An AUC of >0.7 was used due to the exploratory nature of the analysis [20].
- Evaluation of risk factors for acute systemic, chronic systemic, and ocular GVHD: Several baseline characteristics were evaluated using logistic regression models to determine whether any were potential risk factors for the development of acute, chronic, or ocular GVHD.
- If oGVHD was diagnosed in only one eye, then the baseline values corresponding to that eye were used for tear osmolarity, TBUT, Schirmer’s and Oxford corneal staining. If oGVHD was diagnosed in both eyes at different times, then the baseline values corresponding to the eye with the earliest diagnosis were used in the analysis. If oGVHD was diagnosed in both eyes at the same time or if the participant was not diagnosed with oGVHD in either eye, then the eye with the worst baseline values was used in the analysis.

## Results

### Baseline characteristics

This study enrolled 40 participants who intended to receive allogeneic HSCT (intended HSCT cohort) and 20 healthy controls. Description of study participants can be found in Table 1. Compared to healthy controls, the intended HSCT cohort had higher baseline median Oxford corneal staining scores, lower tear RANTES, higher tear TNF-α, higher serum IL-8, and higher serum TNF-α (Table 2). The remaining ocular factors and tear and serum biomarkers were not significantly different between the two groups at baseline (Supplementary Table S1).

**Table 2:** Baseline ocular surface indicators and biomarkers (with significant differences) in the intended HSCT cohort vs. controls.

	Intended HSCT Cohort		Controls		
	N^a^	Median (Range)	N^a^	Median (Range)	Wilcoxon *p* value
Ocular Surface Indicators					
OSDI	40	2.1 (0.0, 37.5)	12	4.2 (0.0, 12.5)	0.51
Oxford Corneal Staining Score	76	1.7 (0.0, 8.7)	40	0.0 (0.0, 1.8)	<0.0001
Schirmer’s Test (mm)	78	10.0 (3.0, 35.0)	38	11.0 (2.0, 35)	0.41
Tear Osmolarity (mOsm/L)	76	305 (284, 378)	40	301 (281, 352)	0.06
TBUT (seconds)	74	8.0 (2.3, 13.0)	38	8.0 (3.3, 12.0)	0.80
Significant Tear Biomarkers (pg/mL)^b^					
RANTES	69	70.4 (0.0, 343.0)	39	190.2 (9.8, 628.0)	<0.0001
TNF-α	69	20.0 (1.3, 109.0)	39	11.2 (0.0, 30.5)	<0.0001
Significant Serum Biomarkers (pg/mL)^b^					
IL-8	34	10.2 (3.3, 957)	18	4.5 (0.0, 38.9)	0.0008
TNF-α	34	8.7 (0.0, 18.1)	18	4.2 (1.5, 9.2)	<0.00001
Dry Eye Disease (%)^c^	(15) 37.5%		(5) 25.0%		0.27

^a^N corresponds to the number of participants for OSDI, serum biomarkers, and Dry Eye Disease; otherwise, corresponds to number of eyes.

^b^Non-significant biomarkers are presented in Supplementary Table 1.

^c^Dry Eye Disease was defined according to 2007 TFOS DEWS Report. Of note, this definition was updated in the 2017 TFOS DEWS II Report Executive Summary, which was published after data collection was completed.

*HSCT* hematopoietic stem cell transplant, *OSDI* ocular surface disease index, *TBUT* tear break-up time.

Four participants in the intended HSCT cohort did not receive the planned transplant and were excluded from subsequent analyses. Two HSCT recipients received no ophthalmological follow-up after transplant due to death. Of the remaining 34 participants (i.e., the analytic cohort), thirty participants received at least 12 months of follow-up; 21 received at least 2 years of follow up, and 16 received at least three years of follow-up (Supplementary Table S2).

### Rates of GVHD

oGVHD was diagnosed in 21 (62%) of the analytic HSCT cohort (Table 3). Most who developed oGVHD did so within the first 12 months (19 [91%]), with a mean time to diagnosis of ~6 months. Chronic GVHD (cGVHD) was diagnosed in 24 (71%) of the analytic HSCT cohort (Supplementary Table S3). The most commonly affected initial organ was the eye (14, [58%]) followed by the mouth (7, [29%]).

**Table 3:** Development of ocular GVHD.

Developed oGVHD	21/34 (61.8%)
Time to diagnosis	
<6 months	8 (38.1%)
6–12 months	11 (52.4%)
>12 months	2 (9.5%)
Severity of oGVHD at time of diagnosis^a^	
1	19 (90.5%)
2	1 (4.8%)
3	1 (4.8%)
Maximum severity of oGVHD at any timepoint^a^	
1	14 (66.7%)
2	5 (23.8%)
3	2 (9.5%)
Organs involved^b^	
Eye only	6 (28.6%)
Eye and others	15 (71.4%)

^a^Severity is based on Ocular NIH Organ Scores calculated according to the NIH Consensus Development Project on Criteria for Clinical Trials in Chronic Graft-versus-host Disease 2014 Diagnosis and Staging Working Group Report.

^b^Organs involved refers to the entire follow-up period; not just time of initial diagnosis.

The global NIH cGVHD score at the time of oGVHD diagnosis mirrored the initial ocular score in most cases, consistent with the finding that the eye was the first organ affected for most participants who developed cGVHD (Table 4A). Examination of the cross-frequencies between the maximum global scores and the maximum ocular score revealed that two of the four cases of severe cGVHD were driven by severe oGVHD (Table 4B). Four of the twelve cases of moderate cGVHD were driven by moderate ocular involvement.

**Table 4:** Ocular and systemic chronic GVHD: cross-frequencies of severity.

A.					
		cGVHD Severity Score at Time of oGVHD Diagnosis			
		1	2	3	Total
oGVHD Severity Score at Time of Diagnosis	**1**	13	5	1	19
		Eye only (11) Skin (1) Genital (1)	Skin (2) Mouth (1) Liver (1) Genital + Mouth (1)	GI (1)	
	**2**	0	1	0	1
			Eye only (1)		
	**3**	0	0	1	1
				Eye only (1)	
	Total	13	6	2	21

B.					
		Maximum cGVHD Severity Score			
		1	2	3	Total
Maximum oGVHD Severity Score	**0**	1	2	0	3
		Mouth (1)	Mouth + GI (1) Skin (1)		
	**1**	7	6	1	14
		Eye only (4) Skin (1) Mouth (1) Genital + Skin (1)	Liver (1) GI (1) Skin (1) Skin + Mouth (1) Joints/ Fascia (1) Skin + GI (1)	GI (1)	
	**2**	0	4	1	5
			Eye only (2) Mouth (2)	Mouth (1)	
	**3**	0	0	2	2
				Eye only (2)	
	Total	8	12	4	24

Cross-Frequencies of **A** Ocular Graft-versus-Host Disease (oGVHD) Severity Score as represented by the Ocular NIH Organ Score and Chronic Graft-versus-Host Disease (cGVHD) Severity Score as represented by the Global NIH Score at Time of Initial oGVHD Diagnosis, **B** Maximum oGVHD Severity Score and Maximum cGVHD Severity Score. Organs listed represent initial organ affected. *GI* Gastrointestinal.

### Ocular surface factors and biomarkers in ocular GVHD

Compared with baseline values, patients who developed oGVHD had lower Schirmer’s test and increased Oxford corneal staining score at time of diagnosis; this was expected as these were defining features of the diagnosis (Table 5). In addition, TBUT was significantly decreased at time of diagnosis of oGVHD compared with baseline (Table 5). OSDI and tear osmolarity were not significantly different. None of the tear and serum biomarkers were significantly different between baseline and time of oGVHD diagnosis (Supplementary Table S4).

**Table 5:** Ocular GVHD cohort: comparison of ocular surface indicators at time of diagnosis vs. baseline.

	N^a^	oGVHD at Baseline	oGVHD at Diagnosis	Wilcoxon *p* value
		Median (Range)	Median (Range)	
Ocular surface parameters				
OSDI	10	2.1 (0.0, 12.5)	2.5 (0.0, 44.0)	0.47
Oxford Corneal Staining Score	23	1.8 (0.1, 5.1)	4.6 (0.4, 9.8)	0.0001
Schirmer’s Test (mm)	23	10.0 (3.0, 25.0)	3.0 (0.0, 10.0)	<0.0001
Tear Osmolarity (mOsm/L)	23	308.0 (296.0, 345.0)	309.0 (288.0, 343.0)	0.86
TBUT (s)	23	9.0 (2.7, 13.0)	4.7 (2.3, 9.7)	0.0004

^a^N corresponds to the number of participants for OSDI; otherwise, corresponds to number of eyes.

OSDI ocular surface disease index, TBUT tear break-up time.

### Receiver operating characteristic (ROC) curve analysis

**Fig. 1:**
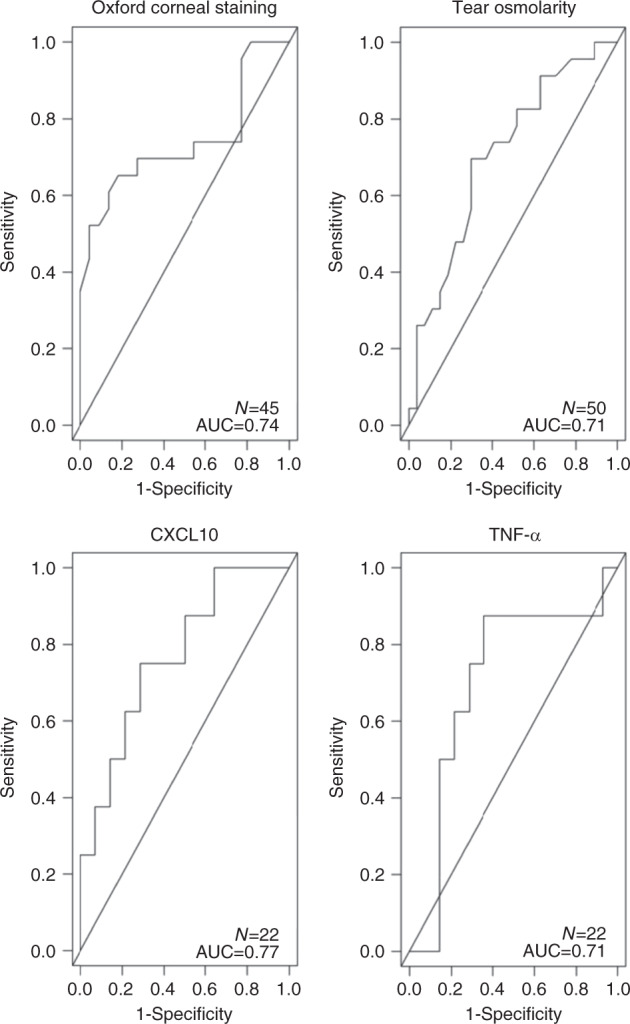
Receiver operating curves for ocular factors and biomarkers with an area under the curve > 0.7. Top left: Oxford Corneal Staining, top right: Tear Osmolarity, bottom left: CXCL10, bottom right: TNF-α.

Among all parameters evaluated, Oxford corneal staining score, tear osmolarity, and serum biomarkers CXCL10 and TNF-α had an AUC > 0.7 for oGVHD diagnosis (Fig. 1). The optimal cutoffs, sensitivities, specificities, Positive Predictive Values (PPV), and Negative Predictive Values (NPV) are presented in Supplementary Table S5.

### Trends in the HSCT Group in the first 12 months

**Fig. 2:**
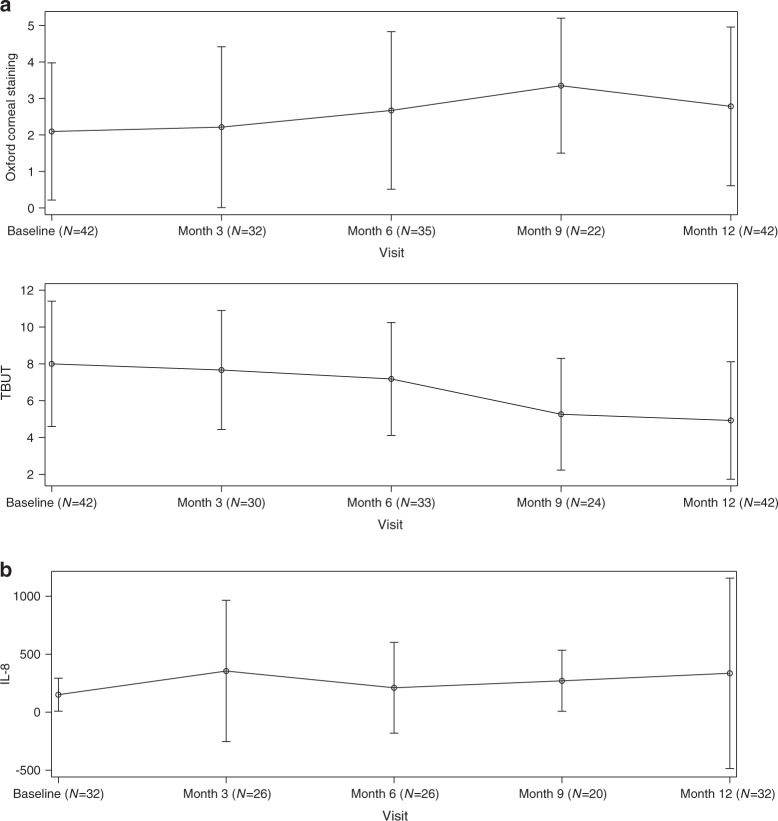
Significant ocular surface parameters and biomarkers over time (Means). **a** Ocular surface parameters. Top: Oxford corneal staining per the Oxford grading scheme as detailed in the methods section, and bottom: Tear Break Up Time (TBUT) in seconds. **b** Tear biomarker Interleukin 8 (IL-8) in pg/mL.

The HSCT cohort was examined longitudinally, comparing ocular surface factors and biomarkers at 12 months to baseline (Supplementary Table S6). Oxford corneal staining score increased and TBUT decreased. When assessed nonparametrically, median tear IL-8 was significantly increased. Trends in these variables over 12 months of follow-up are shown in Fig. 2a, b.

### Risk factors for GVHD

Older age (>40 years) was found to increase the odds of developing oGVHD in the unadjusted model (OR 7.3, *P* = 0.01). Older age was also found to significantly increase the odds of developing cGVHD in both models (Supplementary Table S7). A myeloablative conditioning regimen appeared to moderately increase the odds of developing acute GVHD (aGVHD) compared to a non-myeloablative conditioning regimen in the unadjusted model. No other baseline characteristics significantly increased the odds of developing ocular, acute systemic, or chronic systemic GVHD.

## Discussion

This study demonstrates significant differences in dry eye indicators and selected tear and serum biomarkers in patients with hematological diseases planning to undergo allogeneic HSCT compared to healthy controls. Approximately 38% of HSCT patients demonstrated clinically significant DED at their baseline visit compared to 25% of controls, consistent with previous studies [7, 8]. The HSCT group differed significantly from healthy controls with respect to corneal staining score. Although there has been some debate surrounding how sensitive corneal staining is for DED [21], it provides a robust method for quantifying epithelial surface damage [22]. This damage may be due to the combined effects of conditioning regimens, chemotherapy, immunosuppressive therapy, the hospital environment and other factors not accounted for. We observed a trend toward increased tear osmolarity at baseline in the HSCT group. These findings emphasize the importance of baseline ophthalmologic evaluations of patients prior to HSCT. Diagnosing oGVHD and distinguishing it from DED in the months following HSCT can be challenging if baseline evaluations are unavailable [15, 23]. DED is also known to occur in the majority of patients with cGVHD, regardless of whether they meet strict criteria for oGVHD [23, 24].

An additional challenge in diagnosing oGVHD is the existence of multiple diagnostic and severity scoring criteria. In 2014 the NIH Consensus Development Project removed Schirmer’s testing from organ severity scoring due to poor correlation with symptom change over time [3]. Minimal NIH criteria for diagnosing oGVHD retained Schirmer’s testing, but no longer required distinctive manifestations of cGVHD in another organ [3]. We used the NIH criteria for this study, allowing us to compare findings with other recent studies that also used these criteria [8, 10, 25].

Sixty two percent of HSCT patients developed oGVHD, consistent with a previous NIH-based study [26]. The incidence of oGVHD in the literature ranges from 12-55% [8, 10, 25, 27]. We report a significant decrease in TBUT in those who develop oGVHD, consistent with other studies [10, 27]. Schirmer’s and corneal staining scores have been reported as sensitive markers of oGVHD in multiple studies, as in ours, but carry the same qualifier of commonly being part of the defining criteria [10, 26, 27]. Oxford corneal staining had the highest Positive Predictive Value (79%) for diagnosis of oGVHD at a cutoff value of 3.2. Some groups have shown a strong correlation between increased tear osmolarity and oGVHD [10, 28], while others have failed to show a relationship [29, 30]. Although there was no significant difference in mean tear osmolarities at time of oGVHD diagnosis compared with baseline in our study, ROC analysis rendered a cut-off value of 306 mOsm/L, suggesting that tear osmolarity could contribute to oGVHD diagnosis. No significant difference was observed in OSDI scores between intended HSCT patients and controls at baseline, and OSDI scores did not increase significantly from baseline to time of diagnosis of oGVHD. Of note, OSDI often poorly correlates with clinical signs of DED due to changes in ocular surface sensitivity [21]; despite these limitations, some groups have shown a positive correlation between OSDI and objective measures of oGVHD [15, 27].

Previous studies have shown that risk factors for cGVHD include prior history of aGVHD, older age, female donor to male recipient, mismatched or unrelated donors, donor lymphocyte infusion (DLI), and use of peripheral blood stem cells [31, 32]. In this study, older age was associated with increased odds of developing oGVHD and cGVHD, and a myeloablative conditioning regimen increased the odds of developing aGVHD compared to a non-myeloablative regimen. Other baseline risk factors did not show statistically significant associations, possibly due to the small sample size or differences in study populations. Interestingly, presence of abnormal baseline ocular surface parameters could not predict subsequent development of oGVHD, a finding that may have practical implications if confirmed in future studies.

DED is an inflammatory condition that involves cytokine production by the lacrimal gland [6, 33]. As a result, cytokine and matrix metalloproteinase levels in tears have been investigated in the hopes of discovering non-invasive, sensitive, and predictive markers of oGVHD. Methods of tear collection, assays, and software used for analysis differ among studies, making it challenging to compare results. Ranges of reported values vary considerably, and there are few population-based studies forming established normal levels. Tear fluid MMP-9 is an established marker of ocular surface inflammation [25, 34–36]. It was found to be elevated in the oGVHD group at time of diagnosis and in the HSCT group overall at 12 months post-transplant, though with significant variability so no p-values could be calculated. This study also demonstrated an increase in tear and serum CXCL10 (previously called IP-10) in the oGVHD group at time of diagnosis and in the HSCT group overall at 12 months post-transplant; again, p-values were not calculated due to the wide range of values. Others have shown CXCL10 to predict oGVHD [17]. Tear IL-8 was significantly increased at baseline and at 12 months post-transplant but did not reach statistical significance in the oGVHD cohort and may be elevated in hematologic disorders in general.

Though most studies focus on tear biomarkers due to their proximity to the tissue of interest and ease of procurement, we also examined serum biomarkers in order to compare systemic against organ-specific responses. Serum TNF-α had the highest sensitivity (88%) and NPV (90%) for oGVHD of all the biomarkers studied. Tear and serum TNF-α were significantly higher in the intended HSCT group at baseline compared to the control group, so these also may be elevated in hematologic disorders in general and would require validation as oGVHD-specific biomarkers. TNF-α is a pro-inflammatory cytokine that promotes T cell activation [37], and has been found to correlate with ocular surface parameters and oGVHD severity in other studies [21, 38]. Serum MIP-1α and IL-10 were increased in the oGVHD group at time of diagnosis. These are additional candidates for future investigation.

Limitations of the present report include small sample size, an incomplete data set due to gaps in follow-up of the sickest patients, and an inability to collect tear samples in the most severe cases of oGVHD. These problems also reflect real-world clinical practice. Due to the small sample size, the results from the analyses performed are considered exploratory and not conclusive; the results may be used to inform hypotheses of future studies. Considerable variability and wide confidence intervals were observed in the results presented, which can be attributed to the limited sample size. Strengths of this report include prospective longitudinal study design, comparison of key ocular surface examination indicators with a pre-transplant baseline, and baseline comparisons against a control group. We examined ocular surface indicators, tear and serum biomarkers, and well-annotated clinical transplant and systemic GVHD data at predetermined time points [39]. We show that there are major alterations in the ocular surface and biomarkers prior to HSCT, underscoring the importance of obtaining a baseline eye examination in these patients. This study highlights the importance of TBUT as a clinical marker of oGVHD, in addition to the defining criteria of Oxford corneal staining and Schirmer’s testing. These clinical findings contribute to a large body of research whose limitations include differing defining criteria for oGVHD across studies. Biomarkers hold potential to improve the accuracy of diagnosing oGVHD and better tracking response to treatment. In our study, no specific biomarkers were associated with the development of oGVHD at a statistically significant level. However, certain biomarkers showed possible correlation with oGVHD including tear and serum MMP-9 and CXCL10; tear IL-8; and serum TNF-α, IL-10, and MIP-1α. Each of these are known markers in the GVHD inflammatory pathway and have been linked to DED or to oGVHD in prior studies. Together with ocular surface indicators, these biomarkers represent potential targets for future clinical research.

## Supplementary information

Supplementary Tables
