# Supplementary material for: Ocular surface indicators and biomarkers in chronic ocular graft-versus-host disease: a prospective cohort study
Source: Bone Marrow Transplant. 2021 Mar 8;56(8):1850–8. doi: 10.1038/s41409-021-01254-5 (PMC8338548; doi:10.1038/s41409-021-01254-5)
Supplement: Supplementary file 1 — Supplementary Tables [file 41409_2021_1254_MOESM1_ESM.docx]

**Supplementary Table 1.** Baseline Biomarkers (with Non-Significant Differences) in the Intended HSCT Cohort vs. Controls

|  | **Intended HSCT Cohort** | | **Controls** | |  |
| --- | --- | --- | --- | --- | --- |
|  | N^1^ | Median (Range) | N^1^ | Median (Range) | Wilcoxon p-value |
| **Tear Biomarkers (pg/mL)^2^** | | | | | |
| IFN-γ | 69 | 46.3 (1.7, 313) | 39 | 54.3 (0.0, 159) | 0.42 |
| IL-10 | 69 | 36.8 (3.6, 515) | 39 | 44.8 (0.0, 110) | 0.96 |
| MMP-9 | 69 | 75.2 (2.4, 4941) | 39 | 224.5 (10.6, 44000) | * |
| IL-12 | 69 | 102.9 (2.6, 699) | 39 | 89.0 (0.0, 257) | 0.14 |
| IL-13 | 69 | 34.9 (0.9, 331) | 39 | 47.7 (0.0, 131) | 0.68 |
| IL-17α | 69 | 14.2 (0.0, 162) | 39 | 16.0 (0.0, 51.4) | 0.05 |
| IL-1β | 69 | 11.3 (0.0, 132) | 39 | 15.7 (0.0, 40.6) | 0.36 |
| IL-2 | 69 | 25.1 (0.0, 194) | 39 | 21.1 (1.1, 63.9) | 0.05 |
| IL-4 | 69 | 53.4 (0.0, 876) | 39 | 80.8 (0.0, 260) | 0.004 |
| IL-6 | 69 | 31.0 (1.8, 165) | 39 | 18.8 (0.0, 771) | 0.75 |
| IL-8 | 69 | 82.6 (0.0, 595) | 39 | 90.5 (3.6, 5181) | 0.70 |
| CXCL10 | 69 | 6689 (21.3, 58000) | 39 | 5343 (258, 37000) | * |
| MCP-1 | 69 | 261.3 (4.4, 2233) | 39 | 174.9 (8.1, 5646) | * |
| MIP-1α | 69 | 0.0 (0.0, 16.6) | 39 | 0.0 (0.0, 4.1) | 0.10 |
| **Serum Biomarkers (pg/mL)^2^** | | | | | |
| IFN-γ | 34 | 1.5 (0.0, 34.7) | 18 | 2.4 (0.0, 105) | 0.47 |
| IL-10 | 34 | 9.5 (0.0, 89.6) | 18 | 2.0 (0.0, 426) | 0.07 |
| IL-12 | 34 | 0.0 (0.0, 16.8) | 18 | 1.0 (0.0, 211) | 0.17 |
| IL-13 | 34 | 0.0 (0.0, 104) | 18 | 0.0 (0.0, 12.7) | 0.43 |
| IL-17α | 34 | 1.3 (0.0, 36.7) | 18 | 1.8 (0.0, 51.9) | 0.33 |
| IL-1β | 34 | 0.0 (0.0, 17.4) | 18 | 0.0 (0.0, 5.5) | 0.63 |
| IL-2 | 34 | 0.0 (0.0, 24.2) | 18 | 0.3 (0.0, 6.8) | 0.50 |
| IL-4 | 34 | 0.0 (0.0, 190) | 18 | 0.0 (0.0, 19.6) | 0.04 |
| IL-6 | 34 | 1.5 (0.0, 15.8) | 18 | 0.0 (0.0, 19.6) | 0.02 |
| CXCL10 | 34 | 579.6 (209, 9111) | 18 | 331.0 (24.6, 659) | * |
| MCP-1 | 34 | 546.7 (199, 7504) | 18 | 292.3 (122, 1286) | * |
| MIP-1α | 34 | 4.1 (0.0, 52.8) | 18 | 0.0 (0.0, 1835) | 0.17 |
| RANTES | 34 | 9914 (160, 470000) | 18 | 18906 (3211, 140000) | * |
| MMP-9 | 34 | 34445 (406, 330000) | 18 | 113365.5 (2498, 300000) | * |

HSCT=Hematopoietic Stem Cell Transplant. ^1^N corresponds to the number of participants for serum biomarkers; otherwise, corresponds to number of eyes. ^2^Significant biomarkers are presented in Table 2. *P values are not presented for parameters with considerable variability.

**Supplementary Table 2**. Length of Ophthalmological Follow-Up in the HSCT Cohort (N=34)

| **Follow-up Months** | **Number of Patients** |
| --- | --- |
| **(<1 year)** | 4 (11.8%) |
| 1 | 1 |
| 2 | 1 |
| 8 | 1 |
| 9 | 1 |
| **(1 to <2 years)** | 9 (26.4%) |
| 12 | 4 |
| 14 | 1 |
| 18 | 3 |
| 22 | 1 |
| **(2 to <3 years)** | 5 (14.7%) |
| 24 | 4 |
| 25 | 1 |
| **(3 to <4 years)** | 12 (35.3%) |
| 36 | 7 |
| 38 | 2 |
| 40 | 1 |
| 41 | 1 |
| 42 | 1 |
| **(4 to <5 years)** | 3 (8.8%) |
| 48 | 1 |
| 50 | 1 |
| 52 | 1 |
| **(>5 years)** | 1 (2.9%) |
| 66 | 1 |

HSCT=Hematopoietic Stem Cell Transplant.

**Supplementary Table 3**. Incidence, Severity, and Timing of Chronic and Acute Systemic GVHD in the HSCT Cohort

| **Maximum Global NIH Score for cGVHD**  **N=34** | No cGVHD | 10 (29.4%) |
| --- | --- | --- |
|  | 1 | 8 (23.5%) |
|  | 2 | 12 (35.3%) |
|  | 3 | 4 (11.8%) |
| **Time to initial cGVHD diagnosis (months)**  **N=24** | 1 | 4 (16.7%) |
|  | 2 | 2 (8.3%) |
|  | 3 | 6 (25.0%) |
|  | 4 | 1 (4.2%) |
|  | 6 | 7 (29.2%) |
|  | 9 | 2 (8.3%) |
|  | 15 | 1 (4.2%) |
|  | 18 | 1 (4.2%) |
| Mean (months) | 5 |  |
| **Initial Organ Affected**  **N=24** | Eye | 14 (58.3%) |
|  | Mouth | 7 (29.2%) |
|  | GI | 1 (4.2%) |
|  | Liver | 1 (4.2%) |
|  | Skin | 3 (12.5%) |
|  | Genital | 2 (8.3%) |
| **Maximum aGVHD Grade**  **N=34** | 0 | 10 (29.4%) |
|  | 1 | 6 (17.6%) |
|  | 2 | 6 (17.6%) |
|  | 3 | 8 (23.5%) |
|  | 4 | 4 (11.8%) |
|  | Grades 0-1 | 16 (47.1%) |
|  | Grades 2-4 | 18 (52.9%) |
| **Time to initial aGVHD diagnosis (months)**  **N=24** | 0.5 | 2 (8.3%) |
|  | 1 | 11 (45.8%) |
|  | 2 | 6 (25.0%) |
|  | 3 | 3 (12.5%) |
|  | 4 | 2 (8.3%) |
| Mean (months) | 2 |  |

HSCT=Hematopoietic Stem Cell Transplant, GVHD=Graft-versus-Host Disease. There were multiple organs affected for several participants. Of the analytic HSCT cohort (N=34), 24 had chronic GVHD (cGVHD) and 24 had acute GVHD (aGVHD).

**Supplementary Table 4.** Ocular GVHD Cohort: Comparison of Biomarkers at Time of Diagnosis vs. Baseline

|  |  | **oGVHD at Baseline** | **oGVHD at Diagnosis** |  |
| --- | --- | --- | --- | --- |
|  | N^1^ | Median (Range) | Median (Range) | Wilcoxon p-value |
| **Tear Biomarkers (pg/mL)** | | | | |
| IFN-γ | 18 | 40.2 (11.8, 312.6) | 46.2 (4.9, 205.6) | 0.52 |
| IL-10 | 18 | 30.5 (8.8, 515.5) | 24.5 (2.8, 226.5) | 0.82 |
| MMP-9 | 18 | 96.1 (11.5, 4941) | 211.9 (18.5, 21527) | * |
| IL-12 | 18 | 100.5 (38.8, 699.5) | 108.6 (7.1, 584.2) | 0.36 |
| IL-13 | 18 | 30.7 (9.2, 331.2) | 42.1 (3.2, 207.3) | 0.96 |
| IL-17α | 18 | 9.3 (0.0, 162.0) | 8.6 (0.0, 96.6) | 0.86 |
| IL-1β | 18 | 8.1 (2.6, 131.7) | 10.8 (1.5, 108.2) | 0.32 |
| IL-2 | 18 | 17.3 (8.9, 194.2) | 22.5 (0.7, 117.7) | 0.54 |
| IL-4 | 18 | 36.3 (11.7, 876.4) | 29.3 (0, 636.8) | 0.34 |
| IL-6 | 18 | 23.4 (9.7, 164.6) | 52.8 (9.5, 179.0) | 0.16 |
| IL-8 | 18 | 136.7 (43.1, 595.3) | 296.7 (30.5, 1902) | 0.13 |
| CXCL10 | 18 | 4941.5 (1845, 57878) | 11868 (1033, 59338) | * |
| MCP-1 | 18 | 313.7 (122.4, 1158) | 390.3 (122.4, 1117) | 0.73 |
| MIP-1α | 18 | 0.0 (0.0, 0.0) | 0.0 (0.0, 13.3) | 0.25 |
| RANTES | 18 | 66.7 (23.8, 343) | 98.4 (9.5, 263.4) | 0.96 |
| TNF-α | 18 | 16.8 (8.9, 108.5) | 17.8 (3.5, 102.6) | 0.99 |
| **Serum Biomarkers (pg/mL)** | | | | |
| IFN-γ | 8 | 3.1 (0.0, 17.1) | 2.9 (0.0, 31) | 0.94 |
| IL-10 | 8 | 5.1 (0.0, 89.6) | 10.0 (0.0, 172.3) | 0.03 |
| MMP-9 | 8 | 28141.5 (1278, 182307) | 88648 (13129, 166391) | * |
| IL-12 | 8 | 1.0 (0.0, 3.5) | 0.0 (0.0, 13.5) | 0.56 |
| IL-13 | 8 | 0.0 (0.0, 1.3) | 0.0 (0,.0 71.2) | 0.38 |
| IL-17α | 8 | 0.0 (0.0, 1.9) | 0.9 (0.0, 4.1) | 0.16 |
| IL-1β | 8 | 0.0 (0.0, 0.0) | 0.5 (0.0, 1.6) | 0.13 |
| IL-2 | 8 | 0.5 (0.0, 3.4) | 0.4 (0.0, 3.6) | 0.63 |
| IL-4 | 8 | 0.0 (0.0, 12.9) | 0.0 (0.0, 109.4) | 0.75 |
| IL-6 | 8 | 2.5 (0.0, 11.5) | 1.4 (0.0, 14.2) | 0.84 |
| IL-8 | 8 | 11.3 (3.3, 77.8) | 15.2 (8.2, 75.9) | 0.74 |
| CXCL10 | 8 | 588.2 (389.0, 9111) | 1668.5 (493.4, 6995) | * |
| MCP-1 | 8 | 546.7 (360.7, 1302) | 731.2 (404, 1478) | 0.20 |
| MIP-1α | 8 | 0.7 (0.0, 6) | 6.4 (0.0, 27.1) | 0.05 |
| RANTES | 8 | 8421 (159.5, 36973) | 40831 (1886, 94046) | * |
| TNF-α | 8 | 7.7 (2.8, 18.1) | 18.3 (1.7, 23.6) | 0.05 |

oGVHD=Ocular Graft-versus-Host Disease. ^1^N corresponds to the number of participants for serum biomarkers; otherwise, corresponds to number of eyes. *P values are not presented for parameters with considerable variability.

**Supplementary Table 5.** Optimal Cut-offs for Ocular GVHD Diagnosis using Receiver Operating Curve Analysis

|  |  | **N^1^** | **Cut-off Value** | **Sensitivity**  **(95% CI)**  **(%)** | **Specificity**  **(95% CI)**  **(%)** | **PPV**  **(95% CI)**  **(%)** | **NPV**  **(95% CI)**  **(%)** |
| --- | --- | --- | --- | --- | --- | --- | --- |
| **Ocular Surface Parameters** | Oxford Corneal Staining Score | 45 | 3.2 | 65.2 (48.8, 78.1) | 81.8 (67.9, 92.0) | 79.0 (65.4, 90.4) | 69.2 (53.4, 81.8) |
|  | Tear Osmolarity (mOsm/L) | 50 | 306 | 69.6 (55.4, 82.1) | 70.4 (55.4, 82.1) | 66.7 (51.2, 78.8) | 73.1 (59.7, 85.4) |
| **Serum Biomarkers (pg/mL)** | CXCL10 | 22 | 1091 | 75.0 (49.8, 89.3) | 71.4 (49.8, 89.3) | 60.0 (36.4, 79.3) | 83.3 (59.7, 94.8) |
|  | TNF-α | 22 | 12.3 | 87.5 (65.1, 97.1) | 64.3 (40.7, 82.8) | 58.3 (36.4, 79.3) | 90.0 (70.8, 98.9) |

PPV=Positive Predictive Value, NPV=Negative Predictive Value, CI=Confidence Interval. ^1^N corresponds to the number of participants for serum biomarkers; otherwise, corresponds to number of eyes.

**Supplementary Table 6.** Longitudinal HSCT Cohort: Comparison of Ocular Surface Indicators and Biomarkers at 12 Months Compared with Baseline

|  |  | **HSCT at Baseline** | **HSCT at 12 Months** |  |  |
| --- | --- | --- | --- | --- | --- |
|  | N^1^ | Median (Range) | Median (Range) | Wilcoxon p-value | Longitudinal p-value |
| **Ocular Surface Indicators** | | | | | |
| OSDI | 21 | 2.1 (0.0, 37.5) | 9 (0.0, 44.0) | 0.19 | 0.11 |
| Oxford Corneal Staining Score | 42 | 1.6 (0.0, 8.7) | 2.4 (0.0, 8.7) | 0.07 | 0.02 |
| Schirmer’s Test (mm) | 44 | 9.5 (3.0, 25.0) | 9.5 (1.0, 35.0) | 0.96 | 0.82 |
| Tear Osmolarity (mOsm/L) | 41 | 306 (286, 334) | 302 (285, 330) | 0.13 | 0.13 |
| TBUT (seconds) | 42 | 8.0 (2.3, 13.0) | 3.8 (2.0, 12.0) | <0.0001 | <0.0001 |
| **Tear Biomarkers (pg/mL)** | | | | | |
| IFN-γ | 32 | 51.9 (1.7, 313) | 45.6 (1.7, 530) | 0.97 | 0.64 |
| IL-10 | 32 | 41.6 (3.6, 515) | 33.9 (0.0, 207) | 0.55 | 0.32 |
| MMP-9 | 32 | 80.0 (3.0, 4941) | 126.2 (6.0, 3390) | * | * |
| IL-12 | 32 | 118.2 (2.6, 699) | 85.5 (8.4, 1354) | 0.87 | 0.71 |
| IL-13 | 32 | 34.2 (0.9, 331) | 27.3 (0.0,435) | 0.59 | 0.91 |
| IL-17α | 32 | 19.1 (0.0, 162) | 14.0 (0.0, 91.2) | 0.69 | 0.45 |
| IL-1β | 32 | 11.6 (0.0, 132) | 10.3 (2.2, 146) | 0.32 | 0.86 |
| IL-2 | 32 | 24.8 (0.0, 194) | 20.6 (0.0, 350) | 0.96 | 0.84 |
| IL-4 | 32 | 46.3 (8.6, 876) | 38.9 (0.0, 1235) | 0.78 | 0.98 |
| IL-6 | 32 | 36.6 (1.8, 165) | 33.9 (8.1, 541) | 0.19 | 0.27 |
| IL-8 | 32 | 85.5 (0.0, 595) | 135.1 (28.0, 4724) | <0.0001 | 0.14 |
| CXCL10 | 32 | 5108 (21.3, 30000) | 11032 (2755, 48000) | * | * |
| MCP-1 | 32 | 276 (4.4, 1861) | 382 (61.0, 1806) | 0.02 | 0.52 |
| MIP-1α | 32 | 0.0 (0.0, 0.0) | 0.0 (0.0, 128) | 0.08 | 0.14 |
| RANTES | 32 | 89.6 (0.0, 343) | 92.1 (19.6, 639) | 0.55 | 0.13 |
| TNF-α | 32 | 22.4 (1.3, 109) | 17.7 (0.0, 241) | 0.55 | 0.79 |
| **Serum Biomarkers (pg/mL)** | | | | | |
| IFN-γ | 10 | 1.5 (0.0, 19.1) | 2.3 (0.0, 28.6) | 1.00 | 0.79 |
| IL-10 | 10 | 4.4 (0.0, 78.3) | 13.4 (0.0, 465) | 0.02 | 0.05 |
| IL-12 | 10 | 1.0 (0.0, 9.4) | 0.8 (0.0, 15.5) | 0.81 | 0.56 |
| IL-13 | 10 | 0.0 (0.0, 1.7) | 0.0 (0.0, 39.4) | 0.38 | 0.37 |
| IL-17α | 10 | 0.0 (0.0, 14.0) | 1.1 (0.0, 4.3) | 0.95 | 0.22 |
| IL-1β | 10 | 0.0 (0.0, 1.5) | 0.0 (0.0, 3.9) | 0.69 | 0.29 |
| IL-2 | 10 | 0.0 (0.0, 2.3) | 0.0 (0.0, 10.5) | 1.00 | 0.35 |
| IL-4 | 10 | 0.0 (0.0, 12.9) | 0.0 (0.0, 113) | 0.81 | 0.28 |
| IL-6 | 10 | 1.6 (0.0, 11.5) | 1.6 (0.0, 76.7) | 0.65 | 0.04 |
| IL-8 | 10 | 13.8 (3.3, 957) | 16 (3.7, 271) | 0.85 | 0.48 |
| CXCL10 | 10 | 526 (339, 2000) | 1107 (293, 3415) | * | * |
| MCP-1 | 10 | 516 (361, 1302) | 743 (423, 1883) | 0.06 | 0.05 |
| MIP-1α | 10 | 2.1 (0.0, 46.5) | 7.3 (3.8, 32.0) | 0.13 | 0.50 |
| RANTES | 10 | 8421 (160, 50000) | 27004 (814, 97000) | * | * |
| TNF-α | 10 | 8.1 (2.8, 16.1) | 13.6 (1.9, 179) | 0.19 | 0.05 |
| MMP-9 | 10 | 28142 (1278, 330000) | 76193 (6532, 280000) | * | * |

HSCT=Hematopoietic Stem Cell Transplant, OSDI=Ocular Surface Disease Index, TBUT=Tear Break-Up Time. ^1^N corresponds to the number of participants for OSDI and serum biomarkers; otherwise, corresponds to number of eyes. *P-values are not presented for parameters with considerable variability.

**Supplementary Table 7.** Risk Factors for the Development of Systemic Chronic and Acute GVHD and Chronic Ocular GVHD

|  | **Chronic Ocular GVHD** | | | | **Chronic GVHD** | | | | **Acute GVHD** | | | |
| --- | --- | --- | --- | --- | --- | --- | --- | --- | --- | --- | --- | --- |
|  | Unadjusted Model | | Adjusted Model* | | Unadjusted Model | | Adjusted Model† | | Unadjusted Model | | Adjusted Model‡ | |
| **Baseline Characteristic** | **OR (95% CI)** | ***p*** | **OR (95% CI)** | ***p*** | **OR (95% CI)** | ***p*** | **OR (95% CI)** | ***p*** | **OR (95% CI)** | ***p*** | **OR (95% CI)** | ***p*** |
| **Age**§ | 7.3 (1.5, 35.1) | 0.01 | 5.9 (0.4, 83.9) | 0.2 | 7.3 (1.6, 34.0) | 0.01 | 9.4 (1.2, 75.7) | 0.04 | 2.5 (0.7, 9.4) | 0.2 | 1.8 (0.4, 8.9) | 0.5 |
| **Gender** (male vs. female) | 0.3 (0.1, 1.4) | 0.1 | 0.4 (0.1, 3.0) | 0.4 | 0.5 (0.1, 1.8) | 0.3 | 0.5 (0.1, 2.9) | 0.4 | 1.0 (0.3, 3.7) | 1.0 | 0.8 (0.2, 3.7) | 0.7 |
| **Type of Transplant** (HLA mismatched vs. matched) | 0.2 (0.0, 1.7) | 0.1 | 0.1 (0.0, 3.6) | 0.2 | 0.1 (0.0, 1.4) | 0.1 | 0.3 (0.0, 4.5) | 0.4 | 0.3 (0.0, 3.1) | 0.3 | 1.1 (0.1, 16.7) | 1.0 |
| **Total Body Irradiation** (≥6 Gy vs. <6 Gy) | 1.5 (0.4, 5.7) | 0.6 | 0.2 (0.0, 2.6) | 0.2 | 1.6 (0.4, 6.0) | 0.5 | 0.3 (0.0, 2.0) | 0.2 | 3.1 (0.8, 12.3) | 0.1 |  |  |
| **Conditioning Regimen** (myeloablative vs. non-myeloablative) | 1.3 (0.3, 5.5) | 0.7 |  |  | 1.4 (0.4, 5.6) | 0.6 |  |  | 4.1 (1.0, 16.6) | 0.05 | 2.9 (0.6, 14.5) | 0.2 |
| **Stem Cell Source** (cord and peripheral blood from related haplo donor vs. peripheral blood) | 0.2 (0.0, 1.7) | 0.1 | 1.0 (1.0, 1.0) | 1.0 | 0.1 (0.0, 1.4) | 0.1 | 1.0 (1.0, 1.0) | 1.0 | 0.3 (0.0, 3.1) | 0.3 | 1.0 (1.0, 1.0) | 1.0 |
| **Dry Eye Disease** (yes vs. no) | 0.5 (0.1, 2.1) | 0.4 | 0.4 (0.0, 6.2) | 0.5 |  | | | |  | | | |
| **Tear Osmolarity** (> 308 mOsm/L vs. ≤ 308) | 3.0 (0.7, 12.6) | 0.1 | 1.0 (0.1, 13.4) | 1.0 |  |  |  |  |  |  |  |  |
| **TBUT** (< 10 sec. vs. ≥ 10) | 0.9 (0.2, 4.6) | 0.9 | 0.5 (0.0, 8.9) | 0.6 |  |  |  |  |  |  |  |  |
| **OSDI** (> 13 vs. ≤ 13) | 0.2 (0.0, 1.2) | 0.08 | 0.1 (0.0, 2.0) | 0.1 |  |  |  |  |  |  |  |  |
| **Schirmer’s Test** (< 5mm vs. ≥ 5mm) | 0.9 (0.2, 4.9) | 0.9 | 1.5 (0.1, 31.0) | 0.8 |  |  |  |  |  |  |  |  |
| **Schirmer’s Test** (< 10mm vs. ≥ 10mm) | 1.0 (0.3, 3.9) | 1.0 |  |  |  |  |  |  |  |  |  |  |
| **Oxford Corneal Staining Score** (> 1 vs. ≤ 1) | 1.6 (0.2, 13.3) | 0.6 | 1.1 (0.1, 21.2) | 0.9 |  |  |  |  |  |  |  |  |

OR=Odds Ratio, CI=Confidence Interval, HLA=Human Leukocyte Antigen, Gy=Gray, TBUT=Tear Break-Up Time, OSDI=Ocular Surface Disease Index.

*Model adjusted for all baseline characteristics presented in this table except Schirmer’s (< 10 vs. ≥ 10) and conditioning regimen.

†Model adjusted for all baseline characteristics presented in this table except conditioning regimen.

‡Model adjusted for all baseline characteristics presented in this table except total body irradiation.

§Median age; for cGVHD, >40.5 vs. ≤40.5 years; for aGVHD >42 vs. ≤42 years; for oGVHD >40 vs. ≤40 years based on statistical modeling.
